# Effect of Extremely Low-Frequency Pulsed Electromagnetic Field Intensity and Exposure Time on *Pseudomonas aeruginosa*: An In Vitro Study

**DOI:** 10.3390/microorganisms14040894

**Published:** 2026-04-16

**Authors:** Amal M. El Sawy, Fahda N. Algahtani, Reem Barakat, Aly F. Mohamed, Yosef T. Aladadi

**Affiliations:** 1 Department of Clinical Dental Sciences, College of Dentistry, Princess Nourah bint Abdulrahman University, P.O. Box 84428, Riyadh 11671, Saudi Arabia; amalsawy@pnu.edu.sa (A.M.E.S.); fnalgahtani@pnu.edu.sa (F.N.A.); 2 Dental Clinics Department, King Abdullah bin Abdulaziz University Hospital, Princess Nourah bint Abdulrahman University, P.O. Box 84428, Riyadh 11671, Saudi Arabia; 3 Production of Vaccines, Sera and Drugs, Vacsera, Agouza, Giza 12611, Egypt; fahmy.aly@gmail.com; 4 Department of Electrical Engineering, College of Engineering, Imam Muhammad Ibn Saud Islamic University, Riyadh 11432, Saudi Arabia; ytaladadi@imamu.edu.sa

**Keywords:** bactericidal effect, extremely low-frequency pulsed electromagnetic field, magnetic flux density, noninvasive bactericidal strategy, *Pseudomonas aeruginosa*

## Abstract

Pulsed electromagnetic fields (PEMFs) may exert antimicrobial effects, which could be relevant both in medical applications and as a contributing factor in electro-disinfection processes. This study was designed to evaluate their impact on the viability of *Pseudomonas aeruginosa* (ATCC 27853). Experiments were performed in three independent biological replicates, each with three technical replicates per group. Groups 1–3 served as controls and were not exposed to PEMFs. Groups 4–6, 7–9, and 10–12 were exposed to PEMFs of 40, 60, and 80 µT, respectively, for 4, 8, and 24 h using a cylindrical copper solenoid coil. Bacterial viability was assessed via colony-forming unit (CFU) counts, and log_10_ CFU/mL values were reported. Transmission electron microscopy (TEM) was used to examine structural changes in bacterial cells. PEMF exposure significantly reduced *P. aeruginosa* viability, with magnetic field strength (*p* < 0.001), exposure time (*p* < 0.01), and their interaction (*p* < 0.05) showing significant effects. Post hoc analysis revealed that higher field strengths, particularly 80 µT after 24 h, produced the greatest reduction in CFU counts, whereas 40 µT showed no significant difference compared to controls (*p* > 0.05). TEM images demonstrated pronounced degeneration and structural damage in PEMF-exposed bacterial cells. PEMF exposure reduced CFU counts in an intensity and duration-dependent manner. While a dose-related trend is suggested, limited experimental conditions preclude definitive conclusions, and findings should be interpreted cautiously due to the in vitro design.

## 1. Introduction

The biological effects of pulsed electromagnetic fields (PEMFs) have attracted significant attention globally, particularly in medicine [1]. Research has revealed an intricate relationship between extremely low-frequency PEMFs (ELF-PEMFs) and living systems, with positive effects on human health observed [1]. Exposing cells or microorganisms to ELF-PEMFs can result in intricate interactions involving molecular dipoles, ion channels, and inorganic ions within biological structures. These interactions can modify cell membrane permeability and induce subsequent ionic alterations, suggesting potential applications of ELF-PEMF in therapies and sterilization processes. Notably, ELF-PEMFs can modulate microbial behavior, with the nature and extent of their effects determined by bacterial species, field intensity, frequency, and application duration [2,3,4].

The effects of PEMFs on bacterial viability have been investigated across diverse species, including *Escherichia coli*, *Staphylococcus aureus*, and *Klebsiella pneumoniae*, using a wide range of intensities [5,6,7]. One study reported that extremely low-frequency electromagnetic fields (ELF-EMFs) altered the physicochemical properties of bacterial membranes [8]. Another study reported that ELF-EMF reduced the growth rate of both Gram-positive and Gram-negative bacteria and induced morphological changes [9]. However, a further study found that ELF-EMFs at intensities below 10 µT did not affect bacterial strains such as *E. coli*, *Proteus vulgaris*, *Photobacterium phosphoreum* and *fisheri* [10]. Indeed, bacterial growth in response to electromagnetic fields (EMFs) has also been reported to be strain-dependent and influenced by both exposure time and field intensity [11]. Such variation in experimental conditions makes it difficult to establish standardized antimicrobial parameters.

However, limited data are available regarding the optimal intensity and exposure time required for potential clinical applications [12,13]. Based on the International Commission on Non-Ionizing Radiation Protection guidelines, the European Union has established magnetic field exposure limits at 100 µT for the general public and 200 µT for occupational exposure [6,14,15]. Within this safety framework, there is growing interest in identifying the minimum effective EMF intensity capable of exerting antimicrobial effects, particularly against antibiotic-resistant microorganisms, while remaining within clinically acceptable exposure limits. Moreover, the role of ELF-EMF as a contributing factor in microbial disinfection processes is often overlooked, even though it can influence microbial responses.

*Pseudomonas aeruginosa* is an opportunistic Gram-negative bacterium distinguished by its extraordinary adaptability and resistance to treatment. In humans, it infects the blood, lungs, and other organs [16,17], particularly in those with compromised immune defenses [18]. *P. aeruginosa* biofilms are difficult to eradicate, even with extremely high doses of antibiotics [16,17]. Patients with periodontitis were reported to have higher levels of *P. aeruginosa* within their epithelial cells. Notably, 29 strains of *P. aeruginosa* were isolated from patients with oral infections associated with apical periodontitis and alveolitis in maxillary and mandibular teeth, as well as peri-implantitis [19,20,21]. Thus, there is an urgent need to explore alternative therapeutic strategies beyond conventional antibiotics [22,23].

Exposure to ELF-PEMFs may represent a potential adjunctive approach for diminishing bacterial viability in the management of bacterial infections. The bactericidal effect of EMFs on *P. aeruginosa* has primarily been explored using ELF-EMFs or in conjunction with antibiotics [24,25]. However, a notable gap remains in research on the minimal PEMF dose required to rapidly and effectively eradicate *P. aeruginosa*. Thus, this study primarily aimed to evaluate how PEMF intensity and exposure time influence the growth of *P. aeruginosa*. The null hypothesis was that bacterial cell viability would not differ significantly across varying PEMF intensities and exposure times.

## 2. Materials and Methods

### 2.1. Ethical Considerations and Sample Size

The study received ethical approval from the Institutional Review Board of Princess Nourah bint Abdulrahman University (approval no. 23–0523). The required sample size was estimated using G*Power (version 3.1; Heinrich Heine University, Düsseldorf, Germany), assuming an effect size of 1 [26], a power of 0.95, and a type 1 error rate of 0.05 (5% chance of failing to detect a real effect). The calculation yielded an estimated sample size of 36.

### 2.2. Experimental Design

**Figure 1:**
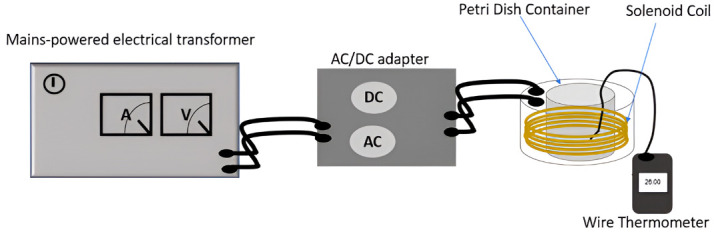
Experimental setup illustration. This illustration displays the experimental setup. A cylindrical copper solenoid coil (187 turns, 16 cm diameter, 10 cm height) generated pulsed electromagnetic fields (PEMFs) via a 220 V/60 Hz main-powered electrical transformer. An AC/DC adapter converts an alternating current (AC) to a controlled pulsed direct current (DC), enabling precise adjustment of the magnetic flux density. A wire thermometer was directly connected to the Petri dish to monitor the temperature.

A specialized device for generating PEMFs was used to investigate their effects on biological structures in *P. aeruginosa* cells (Figure 1). A similar device has been employed in previous in vitro studies [8,27]. To achieve standardized and consistent results, a cylindrical copper solenoid coil (187 turns, 100 m of insulated wire, 16 cm in diameter, 10 cm in height) was used to generate uniform EMFs (Figure 1), allowing for the placement of the bacterial plate in a central position within the cylindrical setup. The cylindrical coil was connected to an electrical transformer, enabling adjustment of PEMF intensity via the voltage (V) and amperage (A) of the alternating current (AC) supply. The electrical transformer (model: LTS602; Thalheime Kühlung GmbH & Co KG, Ellwangen, Germany) operates from a 220 V/Hz AC electrical supply. A 60 Hz controlled-pulsed direct current (DC) generated from a 220 V/60 Hz AC main supply via the electrical transformer and AC/DC adapter was passed through the coil turns, generating a PEMF and yielding target magnetic flux densities of 40, 60, and 80 µT. The PEMF intensity produced by a single turn of the coil can be calculated using the Biot–Savart law [28]:

$$H_{i}=\frac{Ia^{2}}{2{\left(a^{2}+{h_{i}}^{2}\right)}^{3/2}}\;(A/m)$$

In this experiment, distance a is fixed at the radius of the solenoid coil (8 cm); $h_{i}$ is the perpendicular distance from the center of the solenoid to the position of turn i, ranging between 0 and 5 cm (half the height of the solenoid); and I is the controlled pulsed DC. The total magnetic field H is the sum of the 187 individual turns: $H=H_{1}+H_{2}+\ldots +H_{187}$. The magnetic flux density (in T) is given by $B=\mu_{0}H$, where $\mu_{0}$ is the permeability of air (4π × 10^−7^). The applied signal was characterized as a full-wave rectified sinusoidal waveform derived from a 60 Hz AC source, corresponding to a biphasic PEMF. The system’s effective frequency was 60 Hz. The waveform exhibited an approximate duty cycle of 50%, consistent with a rectified signal, and a pulse width of approximately 8.3 ms, corresponding to each half-cycle of the AC waveform. The exposure time was carefully controlled and standardized across all experimental groups to ensure a consistent cycle (~8.3 ms).

To ensure uniformity, EMF intensity was assessed both inside and around the coil surrounding the Petri dish, confirming a consistent EMF distribution across the exposure zone. The magnetic flux density (intensity) was measured via a smart sensor for EMF detection (model: Walfrontxdqsyevwn898; Walfront, Wuhan, China), a portable device with a measurement range of 0–20+ mG, making it suitable for detecting low-frequency EMFs. It operates at frequencies between 50 and 60 Hz, covering both extremely low-frequency and very low-frequency ranges up to 20,000 Hz. In this study, the frequency was set to 60 Hz. The strength of the applied EMF was directly proportional to the current adjustment through the electrical transformer.

A wire thermometer was positioned inside the solenoid coil to monitor temperature variations, preventing overheating and maintaining precise experimental conditions. Throughout the experiments, temperature was monitored to ensure that it did not exceed 40 °C using a digital thermometer with a wire sensor inserted inside the cylindrical coil. Both the control and experimental groups were subjected to identical uncontrolled environmental conditions, which were deemed negligible for this study.

All groups were conducted as independent biological replicates, with experiments performed in separate runs on different days. Environmental conditions, including incubation and handling procedures, were kept consistent across all groups to minimize variability. This design ensured a stable baseline and allowed for isolation of the specific effects of PEMF exposure while reducing potential confounding from environmental factors.

### 2.3. P. aeruginosa Growth

A frozen stock strain of *P*. *aeruginosa, P. aeruginosa* (Schroeter) Migula (ATCC ID: 27853), was used in all experiments. It was grown on tryptic soy agar (TSA) plates (Oxoid Ltd., Basingstoke, UK) at 37 °C. Isolated colonies were inoculated into 5 mL of tryptic soy broth (TSB; Oxoid Ltd., Basingstoke, UK) and grown overnight at 37 °C with vigorous shaking at 180 rpm. After incubation, the cultures were diluted in TSB containing 1% glucose to achieve a standardized optical density at 600 nm corresponding to approximately 1 × 10^8^ colon-forming units (CFU)/mL. Then, tenfold serial dilutions were plated on TSA, and CFUs were counted from two consecutive dilutions that yielded countable plates.

### 2.4. PEMF Exposure

All test plates were positioned at a fixed distance from the coil and centrally aligned at the mid-height of the generator to ensure uniform field exposure. The control groups (Groups 1–3), corresponding to 4, 8, and 24 h, served as time-matched controls and were maintained under the same standardized laboratory conditions as the experimental groups but were not exposed to pulsed electromagnetic fields (PEMFs; 0 µT) within the solenoid coil. In the experimental groups, PEMFs were applied at 40 µT (Groups 4–6), 60 (Groups 7–9), and 80 µT (Groups 10–12) µT. The experiment used time-matched control groups, with Groups 1–3 corresponding to 4, 8, and 24 h, which were maintained under the same standardized laboratory conditions as the experimental groups but were not exposed to a PEMF (0 µT) within the solenoid coil.

The experiment was performed in three independent biological replicates, each comprising three technical replicates per group (a total of nine plates per group). Each plate represented a separate experimental run performed on different days using freshly prepared cultures. All experiments were conducted under consistent laboratory conditions, including standardized incubation temperature and handling procedures, to minimize variability.

### 2.5. CFU Counts

CFU counts were determined via colony counting on the PEMF-exposed (experimental groups) and unexposed (control groups) plates before and after PEMF exposure to assess the effects of PEMF intensity and exposure time. The number of CFUs per plate was obtained from a plate within the linear range of colony counts. These counts were then used to calculate CFUs per milliliter (CFU/mL) based on the sample’s dilution factor. Finally, the CFU/mL values were log_10_-transformed to log_10_(CFU/mL) for reporting.

### 2.6. Evaluation of the Microtexture of P. aeruginosa

A transmission electron microscope (TEM) (JEOL JEM-1010, JEOL Ltd., Peabody, MA, USA) operating at 70 kV was used to examine *P*. *aeruginosa* bacteria-stained sections at the Regional Centre for Mycology and Biotechnology (RCMB), Al-Azhar University. For TEM preparation, bacterial cells were collected by centrifugation (at 4000 rpm for 10 min) from 24 h cultures grown on nutrient broth media and washed with distilled water; the samples were fixed in 3% glutaraldehyde, rinsed in phosphate buffer, and post-fixed in potassium permanganate solution for 5 min, at room temperature. Samples then underwent dehydration and infiltration with epoxy resin. Sections of *P. aeruginosa* bacteria were double-stained in uranyl acetate, followed by 3% lead citrate. Bacterial cellular morphology was assessed to identify alterations in the cell membrane, degeneration or structural deterioration, as indicated by damage to the cell wall and membrane following exposure to PEMFs.

### 2.7. Statistical Analysis

All data analyses were conducted in SPSS (version 20; IBM Corp., Armonk, NY, USA), and *p*-values < 0.05 were considered statistically significant. CFU counts were compared among groups using two-way analysis of variance (ANOVA) followed by post hoc pairwise Tukey’s test to explore the effects of PEMF intensity and exposure time on *P. aeruginosa* growth.

## 3. Results

### 3.1. Morphological Changes

**Figure 2:**
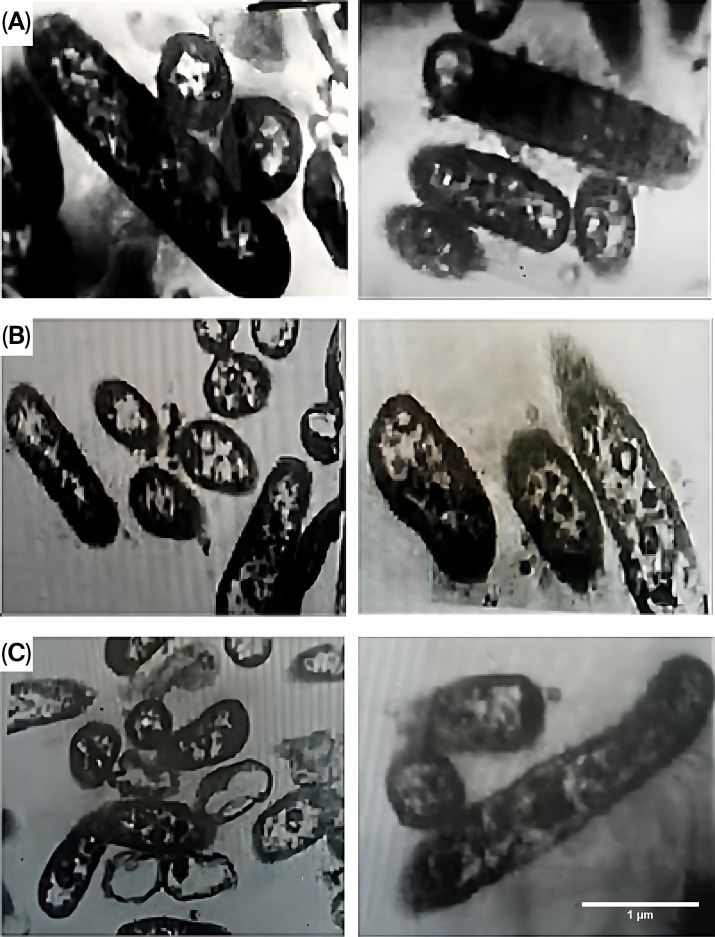
Transmission electron microscope images of *P. aeruginosa*: (**A**) Control group after 24 h. (**B**) *P. aeruginosa* exposed to PEMF at 60 µT for 24 h. (**C**) *P. aeruginosa* exposed to PEMF at 80 µT for 24 h.

In the TEM analysis (Figure 2), the control groups exhibited intact morphology with well-defined cell walls, membranes, cytoplasmic regions containing nucleoid material, ribosomes, plasmids, and surface structures, such as flagella and fimbriae. In contrast, the experimental groups exhibited advanced degeneration and deterioration of cellular structures, as evidenced by damage to cell membranes and the release of cellular content through cracks, leading to deformation and irregular shapes. They also exhibited flattened morphology and contained numerous vacuoles. These morphological changes were attributed to PEMF-induced somatic shock.

### 3.2. Bacterial Viability

**Figure 3:**
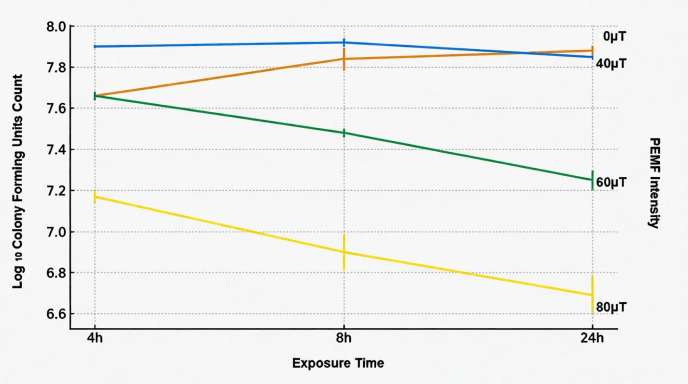
Log_10_ colony-forming unit counts of *P. aeruginosa* in the control and test groups across various exposure settings and durations.

The means and standard deviations of the Log10 CFU counts in the control and test groups are presented in Table 1. The two-way ANOVA analysis revealed that both magnetic field strength (*p* < 0.001) and exposure time (*p* < 0.01) had statistically significant main effects on bacterial growth. Furthermore, there was a significant interaction between magnetic field strength and exposure time (*p* < 0.05), indicating that the effect of the magnetic field depended on the duration of exposure. Post hoc comparisons showed that exposure to higher magnetic field intensities (particularly 80 µT) resulted in a pronounced reduction in bacterial counts compared to the control (0 µT) at all time intervals. The most marked decrease was observed after 24 h at 80 µT (6.69 ± 0.10 log CFU/mL), while non-significant differences were detected between 0 µT and 40 µT groups (*p*> 0.05) (Figure 3).

## 4. Discussion

This study explored the effects of PEMF exposure on the human pathogen *P*. *aeruginosa* at different intensities and exposure times. The findings suggest that higher PEMF intensities, combined with longer exposure times, synergistically reduced *P. aeruginosa* viability and growth; therefore, the null hypothesis was rejected. Understanding the effects of PEMFs on bacteria is crucial for assessing their therapeutic potential and optimizing treatment strategies. The literature suggests promising applications of PEMFs across various fields, including drug design, medicine, pharmacology, and biotechnology. Among the many potential clinical applications of EMFs is the management of injuries and postoperative complications, particularly in promoting bone and soft-tissue healing [29,30,31,32,33].

The time-dependent nature of the PEMF effect on bacterial viability observed in the present study is consistent with findings reported in multiple previous studies. Mohamed et al. (2024) demonstrated that exposing *K. pneumoniae* to a PEMF for durations ranging from 15 to 90 min resulted in progressively greater inhibition of bacterial growth and biofilm formation, with maximum efficacy at 90 min [7]. While another study against *E. coli* and *P. fluorescens* identified exposure duration as one of several critical parameters determining antimicrobial effectiveness [34].

The effects of ELF-EMF on *P. aeruginosa* in the presence of subinhibitory concentrations of antibiotics have also been investigated [35]. While a reduction in *P. aeruginosa* growth was observed after 8 h, consistent with our findings, bacterial adaptation occurred after 24 h, and growth rates became comparable to those of the control group. In the present study, however, exposure to 60 µT and 80 µT resulted in a marked decrease in CFU counts at 24 h. A direct comparison between the two studies is limited by the substantial differences in experimental conditions: the previous study used a single ELF-EMF intensity of 2 mT, whereas our study investigated lower intensities from 40 to 80 µT. In addition, the previous study used antibiotics, introducing a confounding factor that may influence bacterial response to EMF exposure.

Quin et al. studied the viability of *P. aeruginosa* when exposed to ultrasound energy, reporting that high-frequency waves were less effective at reducing bacterial viability than low-frequency waves, and that applying ultrasound energy with a burst power density was equally effective [36]. The effects of ELF-EMF on *E. coli* submerged fermentation cultures have also been examined [37]. Compared with no exposure, EMF exposure changed the viability of *E. coli* cells. Thus, magnetic field generators represent a new and promising approach for recycling cellular suspensions, thereby improving industrial fermentation processes.

Previous research has demonstrated the relationship between magnetic field strength and bacterial inhibition, which was observed in this study. Ahmed et al. reported that the greatest inhibition of *E. coli* growth occurred at the highest tested strength (2.5 mT), while a more recent systematic review concluded that bacterial survival is inversely correlated with field intensity [38]. Sale and Hamilton [39] reported that PEMFs decreased microbial viability at relatively high intensities, with the reduction associated with increased membrane permeability and dependent on both PEMF intensity and exposure time. Similarly, Shoenbach et al. [40] demonstrated that higher PEMF intensities could induce irreversible membrane disruption, leading to cell death due to excessive charge accumulation across the membrane. However, direct comparison with our study is limited, as these studies were conducted at substantially higher PEMF intensities than those applied in our study.

Several mechanisms have been proposed to explain the interaction between PEMFs and microbial systems. EMFs may disrupt cellular functions by inducing vibration of free ions or generating localized charges, potentially leading to DNA damage. Alternatively, EMFs may alter membrane permeability by affecting ion channel function, thereby influencing cellular structure and metabolic activity [41,42,43].

In our study, TEM was used to examine the morphology and viability of *P. aeruginosa*. It revealed advanced degeneration and deterioration of cellular structures, as evidenced by cell membrane damage, loss of cell content (through cracks), deformation, and irregular shape. These findings align with previous studies, reinforcing that EMFs can affect biological tissues and cellular functions. Low-frequency alternating fields can alter cell membrane polarization, potentially exciting or inhibiting electrically active tissues. Some studies have suggested that the power absorbed from EMFs at the mitotic furrow may contribute to the observed antiproliferative effects [44,45,46]. For instance, Beretta et al. reviewed the effects of EMFs on microorganisms from a bioremediation perspective, finding that although some studies have reported positive effects on bacterial activity, others have reported no or sometimes negative effects [47].

The effect of PEMF is dependent on specific physical parameters with effects ranging from stimulatory to inhibitory [34]. A recent study on *E. coli* and *S. aureus* reported that bacterial viability is affected by varying EMF frequencies, further emphasizing the complex role of EMF parameters in determining bacterial growth responses [2].

In our study, *P. aeruginosa* CFU counts were higher at the lowest examined PEMF intensity (40 µT) than in the absence of exposure, suggesting a potential stimulatory effect. This observation is consistent with the concept of a biphasic (dose-dependent) response to EMF exposure, previously reported as hormesis, which describes how exposure may elicit stimulatory effects at lower intensities and inhibitory effects at higher intensities [34,48,49]. In a previous study on *K. pneumonia* biofilm response to ELF-PEMF, biofilm development was reduced after one hour of exposure, while there was a significant increase at 30 min. The authors attributed this temporary rise to an early adaptive response to stress, promoting biofilm formation as a protective strategy [7].

Research has also shown that the biological impact of electromagnetic exposure is highly dependent not only on the specific frequency and field parameters, but also on the intrinsic structural properties of the target microorganism. EMF at a low frequency (50 Hz) enhanced the growth and metabolic activity of Gram-positive bacteria (*S. aureus and Enterococcus faecalis*) by up to 25%, while concurrently inhibiting the proliferation of Gram-negative species (*E. coli*) by up to 17%. This differential response has been attributed to structural and functional differences in cell wall composition and transport mechanisms between the two bacterial groups [50]. Fijałkowski et al. [51,52] examined the effects of PEMFs (34 mT, 50 Hz) on different bacterial species, including *P. aeruginosa*. The effects differed among the bacterial species, with *P. aeruginosa* exhibiting decreased metabolic activity. Thus, they suggested that PEMF exposure may induce the expression of heat shock proteins, which protect against thermal shock and help repair or degrade damaged proteins.

The observed reduction in *P. aeruginosa* viability and growth following exposure to higher PEMF intensities (approximately 1 log_10_ CFU/mL at 80 µT) is modest when compared to conventional disinfection methods, which routinely achieve reductions exceeding 7 log_10_ units [53]. Furthermore, thermal electromagnetic induction heating has been demonstrated to produce a 5–6 log reduction at 60 °C, underscoring the critical antibacterial role of temperature [54]. In light of these comparisons, the effect observed in this study is more appropriately classified as growth inhibition rather than bactericidal activity. This interpretation aligns with previous reports on EMF exposure, which have consistently described alterations in bacterial growth dynamics and metabolic activity without complete eradication [51]. Thus, PEMF is best positioned as an adjunctive strategy that modulates microbial behavior, rather than a standalone disinfection modality.

The ability of *P. aeruginosa* to form robust biofilms, produce a wide range of virulence factors, and employ multiple antimicrobial resistance mechanisms makes its treatment clinically challenging. These traits enable it to persist in hospital environments, resist disinfection, and survive antibiotic therapy. The World Health Organization has labeled *P. aeruginosa* as a critical priority pathogen, emphasizing the urgent need for novel therapeutic strategies [55,56]. With antimicrobial resistance posing a growing global health threat, alternative non-chemical strategies such as EMF have gained attention as potential tools to inhibit bacterial growth. EMF offers a physical method to disrupt microbial viability, providing a complementary approach to conventional antibiotics, particularly against resistant strains [57]. Multi-drug-resistant *P. aeruginosa* infections are associated with high mortality due to limited therapeutic options [58]. In this context, even modest reductions in bacterial load, such as the one observed in this study, may carry clinical relevance, particularly as an adjunctive therapy alongside existing antibiotics.

This in vitro study has several limitations that should be acknowledged, primarily stemming from its simplified design. Experiments were performed in triplicate, resulting in a small sample size that may reduce statistical power and potentially inflate the observed effect size. The study evaluated PEMF effects on a single isolated bacterial species, without considering interactions with other microorganisms or host factors. In clinical settings, bacteria often exist within single- or multi-species biofilms, where microbial interactions and environmental conditions can significantly influence growth and susceptibility. Consequently, the direct extrapolation of these findings to clinical scenarios is limited. The primary implication of this study is not that PEMF represents a clinically validated *P. aeruginosa* inhibitor, but rather that it provides proof-of-principle evidence of bacterial susceptibility to PEMF. Clinical application would be premature without further investigation using validated multi-species biofilm models. Future research should examine PEMF effects in more complex biological environments, accounting for microbial diversity, host immune responses, and tissue microenvironments.

## 5. Conclusions

PEMF exposure was associated with changes in *P. aeruginosa* viability and growth, with higher intensities (60 and 80 µT) producing greater reductions. Increased exposure time was also associated with a more pronounced reduction, particularly at higher intensities. The overall reduction remained modest, indicating PEMF’s potential as an adjunctive modality that alters microbial behavior, rather than a standalone disinfection tool. Within the limitations of the current in vitro study design, the interpretation of the findings should be considered preliminary, serving as a hypothesis for further research to better evaluate its clinical applicability.

## Figures and Tables

**Table 1:** Mean and standard deviation of *P*. *aeruginosa* CFU counts following exposure to varying intensities of PEMF across different durations.

Time	Magnetic Field Strength			
	0 µT	40 µT	60 µT	80 µT
4 h	7.66 ± 0.010	7.90 ± 0.008	7.66 ± 0.020	7.17 ± 0.030
8 h	7.84 ± 0.054	7.92 ± 0.020	7.48 ± 0.020	6.90 ± 0.085
24 h	7.88 ± 0.021	7.85 ± 0.009	7.25 ± 0.045	6.69 ± 0.096

## Data Availability

The original contributions presented in this study are included in the article. Further inquiries can be directed to the corresponding author.

## Abbreviations

The following abbreviations are used in this manuscript:

PEMFs	Pulsed Electromagnetic Fields
ELF_PEMFs	Extremely Low-Frequency Pulsed Electromagnetic Fields
WHO	World Health Organization
IF	Intermediate Frequency
ELF	Extremely Low Frequency
RF	Radio Frequency
